# Impact of Global Warming on the Severity of Viral Diseases: A Potentially Alarming Threat to Sustainable Aquaculture Worldwide

**DOI:** 10.3390/microorganisms11041049

**Published:** 2023-04-17

**Authors:** Marine Combe, Miriam Reverter, Domenico Caruso, Elodie Pepey, Rodolphe Elie Gozlan

**Affiliations:** 1 ISEM, Université de Montpellier, CNRS, IRD, 34095 Montpellier, France; 2 Marine Biology and Ecology Research Centre, School of Biological and Marine Sciences, University of Plymouth, Plymouth PL4 8AA, UK; 3 CIRAD, UMR ISEM, 34398 Montpellier, France

**Keywords:** viral diseases, global warming, one health, sustainable aquaculture, food security

## Abstract

With an ever-increasing human population, food security remains a central issue for the coming years. The magnitude of the environmental impacts of food production has motivated the assessment of the environmental and health benefits of shifting diets, from meat to fish and seafood. One of the main concerns for the sustainable development of aquaculture is the emergence and spread of infectious animal diseases in a warming climate. We conducted a meta-analysis to investigate the influence of global warming on mortality due to viral infections in farmed aquatic animals. We found a positive trend between increasing temperature and increasing viral virulence, with an increase in water temperature of 1 °C resulting in an increase in mortality of 1.47–8.33% in OsHV-1 infected oysters, 2.55–6.98% in carps infected with CyHV-3 and 2.18–5.37% in fishes infected with NVVs. We suggest that global warming is going to pose a risk of viral disease outbreaks in aquaculture and could compromise global food security.

## 1. Introduction

It is estimated that about 2.6 billion people in the developing world have to live on less than USD 2 a day, of whom 1.4 billion are “extremely” poor, i.e., they survive on less than USD 1.25 a day. Nearly three quarters of the extremely poor (1 billion people) live in rural areas. Most rural households depend on agriculture for their livelihoods, and livestock are usually an integral part of their production system. Reducing the pressures of food production on the environment while feeding an ever-increasing human population is one of the major challenges facing humanity. The magnitude of the environmental impacts of food production, mainly in terms of land use, has motivated the assessment of the environmental and health benefits of changing diets, usually from meat to other sources, including fish and seafood. Aquaculture is the farming of aquatic animals or plants, and can range from small-scale natural production (e.g., rice fish farming) to extensive or semi-extensive farming, fertilized or unfertilized (e.g., pond farming), or intensive farming (e.g., off-shore fish cage) and finally to hyper-extensive farming (raceways). It is one of the fastest-growing food production sectors in the world, with low- and middle-income countries (LMICs) accounting for more than 90% of global aquaculture production [1,2]. Importantly, aquaculture contributes significantly to the economy of many households [3], playing a major role in global food security and poverty reduction, which are the main concerns of the United Nations 2030 Agenda for Sustainable Development Goals [4]. However, aquaculture can also have negative impacts on the surrounding environment by increasing stress on water resources, overfishing of wild stocks, introducing invasive species or promoting the emergence and transmission of pathogens [1,5,6]. With the aquaculture sector expected to grow by 62% between 2010 and 2030 to meet the increasing demand for protein consumption [7], there is an urgent need to adopt innovative and sustainable aquaculture practices to enhance ecosystem services, such as wastewater treatment, bioremediation, habitat restoration and the recovery of wild populations [5,8]. The development of sustainable production systems is not only in the UN 2030 Agenda for Sustainable Development, but also in the Rome Declaration of the Second International Conference on Nutrition (ICN2) and the UN Framework Convention on Climate Change (COP21) [2].

With increasing world trade and intensification of production systems, one of the main concerns for the sustainable development of aquaculture is the emergence and transmission of animal infectious diseases [6]. In fact, despite improvements in disease surveillance and management, economic losses due to disease outbreaks in aquaculture are estimated to exceed USD 9.5 billion per year [9]. For example, high-density production systems and warm water temperatures have an impact on water quality (changes in salinity, alkalinity, introduction of pollutants and drug residues, lower oxygen levels), making stressed and immunosuppressed animals more susceptible to infection, and contributing to the spread of virulent strains of pathogens [10,11,12]. Importantly, emerging infectious diseases (EIDs) are expected to increase with warming temperatures [3,13,14,15], and EID outbreaks in aquatic species (pathogens carried by wild animals transmitted to and emerging in farmed animals, or pathogens introduced into new habitats and emerging in the same or different hosts) [1] at lower latitudes may be partly due to higher temperatures and higher nutrient levels [16]. For example, aquatic animals infected with bacterial pathogens show higher mortality at higher temperatures [6]. Therefore, the increase in global average temperature of 1.4–5.8 °C by 2100, as predicted by the UN Intergovernmental Panel on Climate Change (IPCC), is an alarming risk factor for the frequency of EID outbreaks in farmed aquatic animals [13], and poses a serious global health threat in terms of food security. Unfortunately, climate change still remains a largely neglected environmental challenge for the sustainability of the aquaculture sector.

The incidence, frequency and geographic extent of viral infections in farmed aquatic animals tend to increase with climate changes and the expansion of global trade [1], with major economic and social consequences on a global scale. For example, viral pathogens are the most important constraint on the growth and survival of farmed crustaceans, and it is estimated that up to 40% of shrimp production in the tropics is lost each year due to viral infections [17]. Since its outbreak in China and Taiwan between 1991 and 1992, global economic losses caused by the white spot syndrome virus (WSSV), which causes white spot disease (WSD) in shrimp and many other crustaceans worldwide, have been estimated to be in the range of USD 8–15 billion [18]. Similarly, the Tilapia lake virus (TiLV), a new emerging viral disease of fish, caused massive losses of tilapia stocks in Israel and Ecuador in 2009, and has subsequently been responsible for mortality rates ranging from 10 to 90% (depending on the strain) in farmed and wild tilapia across 12 countries in Asia, Africa and South America [1,19,20]. Tilapia is the second farmed fish species after carps, in the world, with a global tilapia trade estimated at over USD 7.5 billion [19], so TiLV is a significant risk to food security [1,20]. Many studies have suggested that environmental factors, such as temperature, salinity, pH and eutrophication, represent stressors that can influence the transmission and occurrence of viral outbreaks [21]. For example, it has been proposed that sudden changes in temperature away from animals’ optimal growth temperature have an impact on all physiological processes (metabolism, oxygen consumption, growth rate, moulting cycle and survival rate) in fish and shrimp, including immunity to viral diseases [22,23]. For example, temperature drops below 32 °C have been associated with higher replication rates and infectivity by WSD and reduced immunity in shrimp [24,25,26,27,28]. In contrast, cyprinid herpesvirus 3 (CyHV-3) and the µVar variant of Ostreid herpesvirus OsHV-1, responsible for koi herpesvirus disease (KHVD) and Ostreid herpesvirus disease (OsHVD) worldwide, respectively, have been associated with increases in water temperature [29,30,31]. Betanodaviruses, also known as viral nervous necrosis (VNN) viruses, cause encephalopathy and retinopathy (VER) in fish in marine environments [32,33]. Some experiments have shown higher virulence (in terms of host mortality) of Betanodavirus in fish (*Solea senegalensis*, *Epinephelus akaara*) exposed to higher water temperatures (100% host mortality at 22 °C vs. 8% at 16 °C) [32,34], whilst others found inhibition of viral replication at higher temperatures [35]. However, Betanodavirus are ubiquitous in marine aquaculture and are responsible for frequent occurrences of VNN disease causing, for example, significant losses in *Cromileptes altivelis* farming in tropical areas [35]. An increased immune response against inactivated lymphocystis disease virus (LCDV) has also been observed in Japanese flounder *Paralichthys olivaceus* with increasing temperature rises [36]. However, to date, few studies have measured comparable and quantifiable biological parameters to estimate the potential impact of global warming on viral infections in farmed aquatic animals, and none have attempted to statistically establish a clear trend between increased water temperatures and virulence (in terms of host mortality rates) of viral diseases.

Finally, it is also important to understand that aquaculture in the vast majority of productions is a production open to other environmental compartments. For example, the water used for aquaculture farms is often that of cities, villages or ponds fertilized with other agricultural products, such as manure from pig or chicken farms. This makes them very vulnerable to the introduction of pathogens from all horizons, which defines them as hot spots for pathogen emergence. The deregulated use of drugs to treat these emerging infections also presents a risk of emergence of resistance to sanitary treatment, leading to serious health problems for animals and humans. It is therefore understandable that aquaculture is at the crossroads of infection risk, food security and global warming [6].

Here we conducted a meta-analysis based on published studies of viral infections in farmed aquatic animals. In order to obtain reliable data on cumulative host mortalities under fixed temperatures, we established a list of inclusion criteria from the literature, including a minimum number of five independent studies on a given viral vector. According to these criteria, only three viral diseases (KHVD, OsHVD and Betanodaviruses) were included in the meta-analysis. However, all are considered to be emerging diseases due to the expansion of their geographical and host range after the first reported outbreaks, and infect a wide variety of organisms (freshwater, brackish and marine fish and shellfish) from different habitats in tropical, subtropical and temperate regions worldwide [32,37,38], therefore being a good model to test whether increased water temperature can increase the severity of viral infections (increased mortality rates) in aquatic animals farmed worldwide.

## 2. Methods

### 2.1. Literature Search and Data Collection

We systematically searched for all peer-reviewed journal articles that studied cultured aquatic animal mortalities due to viral infections, using the Web of Science, up to 1 March 2019. Grey literature (i.e., student thesis and technical reports) was not included in the literature review to prevent bias (i.e., only English grey literature would have been identified in our search). As articles studying viral infections in aquatic animals often use highly specific terminology (i.e., virus or species names, in the title, keywords and topic) we used a two-step literature search. First, we conducted a general search to identify viral infections in aquatic animals, for which we could obtain at least five eligible articles. For this first search, we used the following generic keywords: (fish* OR shrimp* OR oyster* OR mollusc*) AND (mortality OR outbreak OR infection) AND virus AND temperature. We reviewed each article to determine whether it met the following criteria: cumulative mortality and stable specific temperature (+/− 2 °C) were reported for infected aquatic cultured animals. We identified only three viral pathogens (OsHV-1, CyHV-3 and Betanodaviruses), for which we could obtain sufficient data (threshold of 5 independent articles). We then conducted specific searches on these viral infections (up to 27 March 2023) to identify other relevant studies that did not appear in our first general search, using the following keywords: (carp* OR oyster* OR fish* OR shrimp* OR mollusc*) AND (mortality OR outbreak OR infection) AND (KHV OR CyHV-3 OR OsHV-1 OR betanodavirus*). This search yielded 736 articles, which were examined for eligibility. We only retained articles that reported cumulative mortality at a specific temperature. Articles from field reports or experimental trials, where temperature was not fixed (+/− 2 °C), were not included. Only articles where experimental infections were conducted at a specified infective dose and only one pathogen was detected and clearly identified (i.e., through PCR or similar) were included (i.e., multi-infection experiments or articles where secondary infections were identified were excluded). The following data were extracted from each of the selected articles: taxonomy of the pathogen and host (family and species), stage of host development (spats, only for oysters, juvenile, adult), country, temperature of the infection, cumulative mortality and type of infection (injection or experimental by immersion). When a study included several experiments at different temperatures, we considered them as separate observations. We obtained a dataset containing 160 observations (cumulative mortality at specific water temperature) extracted from 53 studies, belonging to three viral pathogens (OsHV-1,CyHV-3 and Betanodaviruses). The list of publications used here are available in the Supplementary Information S1.

### 2.2. Data Analysis

The three datasets (CyHV-3, OsHV-1 and Betanodaviruses) were analysed separately to investigate the effect of temperature on mortality associated with each disease. We also analysed all the results together, to investigate the general trends in the effect of temperature on virus mortality in aquaculture.

Nested linear regression models were constructed to examine the relationship between mortality of virus-infected animals and temperature. The fixed effects included temperature, life stage, type of infection and infection dose (log-transformed). Random effects were only used in the analysis of the Betanodaviruses dataset and overall dataset (i.e., containing CyHV-3, OsHV-1 and Betanodaviruses), and included the host and pathogen taxonomy. Akaike’s Information Criterion for small sample sizes (AICc) was used to assess the explanatory value and parsimony of each model. The difference in AICc values between each model and the best-fitting model with the lowest AICc (ΔAICc) was used to determine the strength of each model. Akaike weights (w_i_), which determine the weight of evidence of each model relative to the set of candidate models, were then used to select the model with the best fit (model with the highest weight) [39] (Supplementary Information S2–S5). Plots of the selected model (residuals, QQ and partial autocorrelation) were explored to detect model assumption violations (Supplementary Information S6).

## 3. Results

### 3.1. Relationship between Mortality of Virus-Infected Animals and Temperature

**Figure 1:**
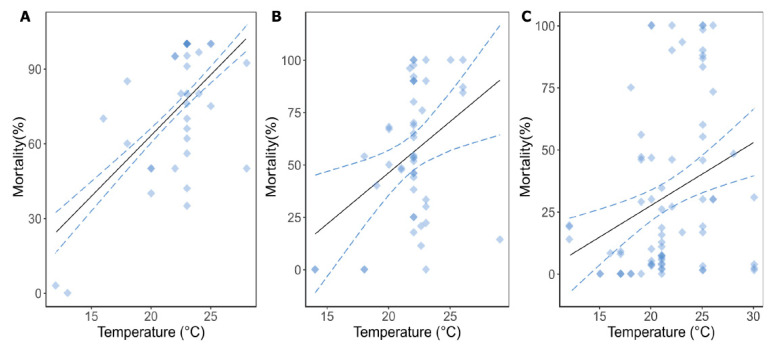
Predicted changes in mortality (%) of reared aquatic animals infected by viral pathogens related to temperature increases. (**A**) Koi Herpesvirus Disease (KHV, CyHV-3, *n* = 36), (**B**) Ostreid Herpesvirus Disease (OsHV-1, *n* = 49), (**C**) Betanodaviruses (NVVs, *n* = 75). Dots represent the raw data extracted from the originally reviewed literature. Solid black lines represent predictions arising from the selected linear and linear mixed models (LM and LMMs, Table 1). The dotted lines represent the 95% confidence intervals of the prediction.

We collected data from 53 experimental studies to investigate the influence of temperature on the mortality of farmed aquatic animals infected with CyHV-3, OsHV-1 and different variants of Betanodaviruses (NVVs). Linear regression models showed that increasing water temperature resulted in higher mortality rates in CyHV-3-infected carp (*Cyprinus carpio*), OsHV-1-infected oysters (*Crassostrea gigas*) and fishes infected by NVVs (Table 1, Figure 1A–C). None of the models’ fixed effects (life stage, type of infection and log(dose)), except temperature, were important predictors for the mortality OsHV-1-infected oysters’ mortality (Supplementary Information S2). In the CyHV-3 model, the type of infection was an important predictor of mortality in carps, and was therefore included in the selected model (Supplementary Information S3). The Betanodaviruses dataset was comprised of different fish species and different variants of Betanodaviruses; therefore, host and pathogen taxonomy (species and family) were added in the models. The model with the best fit included the pathogen and host species as random effects and life stage and type of infection as fixed effects (Supplementary Information S4).

### 3.2. Model Predictions for OsHV-1, CyHV-3 and NVVs

**Figure 2:**
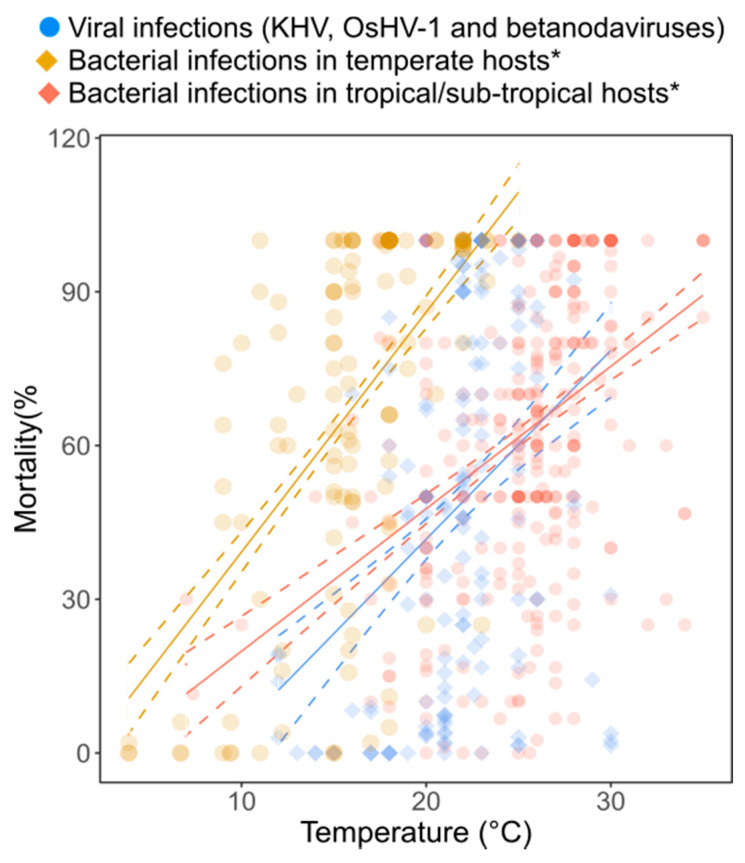
Predicted changes in mortality (%) of reared aquatic animals infected by bacteria * (yellow and red, * data previously published in Reverter et al. 2020) [6] and viral (blue, ostreid herpesvirus, koi herpesvirus and betanodaviruses) diseases in response to temperature. Dots display the raw data retrieved from research articles (n_viruses_ = 160, n_temp. bacteria_ = 129, n_warm bacteria_ = 329). Solid lines represent the predicted mortalities according to selected linear and linear mixed models (Table 1), with dotted lines representing the 95% confidence interval for each of the models.

The models predicted that an increase in water temperature of 1 °C would result in an increase in mortality of 1.47–8.33% (95% confidence interval, CI) in OsHV-1-infected-oysters, 2.55–6.98% (95% CI) in CyHV-3-infected carps and 2.18–5.37% (95% CI) in NVV-infected fishes (Table 1, Figure 1). The linear model combining the three datasets (CyHV-3, OsHV-1, NVVs) showed that an overall increase in water temperature of 1 °C would result in 3.07–5.70% (95% CI) increase in the mortality of these virus-infected animals (Table 1, Figure 2). The selected model for the overall virus dataset included host species as the random effect, and life stage and type of infection as the fixed effects (Supplementary Information S5).

## 4. Discussion

### 4.1. Impact of Global Warming on Viral Epidemics in Aquaculture Systems

While our results highlight the paucity of empirical data available to date on the effects of global water temperature increase and its impact on viral virulence (host mortality rate) in farmed aquatic animals, we did find a positive trend between increased water temperature and increased virulence for CyHV-3, OsHV-1 and NVVs infections. Interestingly, even small increases in water temperature, such as a 1 °C rise, could result in a 3.07–5.70% (95% CI) increase in mortality of animals infected with CyHV-3, OsHV-1 and NVVs. This is 1.09–1.83 times higher than the increases in bacterial mortality expected in warm waters, but slightly lower (1.05–1.26 times lower) than the bacterial-related mortality expected in temperate animals (Figure 2) [6]. To date, the role of increased water temperature on the selection and spread of viral pathogen strains remains controversial. For example, Saker et al. (2004) [13] suggested that viral infections are not enhanced by increasing temperatures and several experimental studies on WSSD in shrimp have shown lower viral replication rates and higher immune responses for increased temperatures [24,25,26,27,28,40,41]. In contrast, Jiravanichpaisal et al. (2004) [42] found that WSSD experimentally infected freshwater crayfishes at the warmest temperatures studied (22 °C) exhibited the highest mortality, and suggested that higher temperatures may support WSSD replication. Other studies on terrestrial systems showed that increases in environmental temperatures can lead to increased viral propagation, both within (i.e., cell-to-cell) and amongst hosts, leading to higher viral loads [43,44]. These studies suggest that the relationship between temperature and viral replication and propagation remains complex [45], often species-specific, and needs to be better characterized. However, evidence suggests that increases in temperature outside the normal range cause cellular stress and weaken the immune system of most aquatic ectotherm species, making them more vulnerable to infection and perhaps mortality [46,47]. Here, we sought to conduct a comprehensive meta-analysis to clarify the role that increased temperature might play in mortality caused by viral infections in cultured aquatic species. Although our meta-analysis focused only on the effects caused by three types of viral pathogens in aquaculture (CyHV-3, OsHV-1 and NVVs), due to the lack of comparable experimental and field data between species and pathogens, thus preventing he inclusion of other pathogens in the analyses, we showed that for these two globally important diseases, a higher temperature leads to increased host mortalities. CyHV-3 is one of the most widespread (from tropical to temperate regions) and important aquatic viral pathogens, causing major epidemics worldwide, with reported mortality rates of 40–100% in koi carps *Cyprinus carpio* [29,30]. Furthermore, previous studies showed that water temperature is a key factor in the (re)activation of CyHV-3 [48,49]. Therefore, it represents a good biological model to investigate its virulence under different temperature regimes. Similarly, OsHVD has been associated with recurrent mass mortality events (up to 100%) in young Pacific oysters, *Crassostrea gigas*, which appear to be linked to abnormally warm water temperatures (above 16–24 °C) in spring and summer [31]. Importantly, the immunosuppression caused by OsHV-1 appears to facilitate secondary bacterial infections, such as those caused by virulent opportunistic *Vibrio* species, which may also be responsible for the mortalities observed in oyster [50,51]. The datasets used here for KHVD and OsHVD include studies in which viral inoculums were experimentally injected into healthy animal hosts, and the presence of bacteraemia following viral infections was not reported. Although unlikely, the presence of secondary bacterial infections cannot be completely excluded, and a potential undetected co-infection could be related to the higher mortality increases (4.06–9.17% increased mortality/°C) observed in OsHVD-infected oysters. Finally, Betanodaviruses are the agents responsible for the highly infectious disease VNN, which results in the necrosis and vacuolation of the brain, retina and spinal cord of infected fish and invertebrates [32,52]. Furthermore, VNN is considered one of the most important threats to fish farming, as it can affect 44 fish species from 24 families worldwide [52]. Whilst some genotypes (BFNNV, TPNNV) cause disease in cold-water fish, other genotypes (RGNNV, SJNNV) infect warm-water species, and some studies have shown increased virulence at higher temperatures (i.e., increased viral load, increased host mortality), although viral infection can still occur at low temperatures [52] and also depending on the genetic characteristics of certain viral genotypes [52]. Nevertheless, overall, this suggests that water temperature plays an important role in determining the onset and severity of disease [52]. Importantly, our results draw attention to the risk of viral disease outbreaks in aquaculture in the context of global warming. Increasing temperatures could not only result in higher mortalities in aquaculture settings, but could potentially lead to the selection and spread of virulent viral strains to wild organisms, as previously observed with KHVD outbreaks [29,30]. As mentioned earlier, the UN Intergovernmental Panel on Climate Change (IPCC) predicts a global average temperature increase of 1.4–5.8 °C by 2100, which will therefore pose a worrying threat to aquaculture sustainability and thus food security. Further research is therefore urgently needed to better understand whether the trends observed here (i.e., increased mortality in animals infected with KHVD, OsHVD and NVVs) are maintained amongst other relevant aquatic viral diseases. Research efforts are equally needed to understand the complexity of co-infection dynamics in the context of global warming.

### 4.2. Sustainable Aquaculture Practices to Prevent EID Outbreaks

The global human population is expected to reach 9.7 billion people [53] by the year 2050, and agricultural practices will therefore be under greater pressure for food production [54]. Although aquaculture can provide public health, economic and social benefits, One Health approaches will be needed in the future to optimize the trade-offs between economic benefits and productivity while considering animal, environmental and human health [2]. In the current context of global warming, global trade and production intensification, public health risk assessment is fundamental. Given the risk of EID, profiling and monitoring the prevalence of (viral) pathogens in aquaculture systems (including water, sediments and animal compartments) in a precautionary manner is inevitable. Surveillance systems should focus on identifying not only the vectors, but also healthy carriers, such as hosts with an extremely low pathogen load and/or absence of clinical signs, but capable of transmitting the pathogen to susceptible hosts [1]. Furthermore, as the presence of a pathogen in a specific environment does not imply a risk of disease emergence, it is necessary to calibrate this surveillance in order to determine the baseline for pathogen circulation (the normal pathogen load in the environment) versus continued pathogen emergence (increase in pathogen load). Such enhanced disease surveillance in aquaculture facilities and closed wild environments, e.g., using environmental DNA (eDNA) surveys, could also avoid the routine prophylactic use of antimicrobials in aquatic farms, especially in LMICs, for diseases caused by viral or fungal pathogens. In addition, these eDNA approaches can identify biosecurity risks that aquaculture farms pose to the surrounding environment, preventing the spread of pathogens to wild animals and vice versa [2]. Overall, such global surveillance would require coordinated, consistent and shared data collection within and between countries, as well as the implementation of effective stock management strategies to minimize the risk of EIDs.

Several sustainable solutions to viral (but also bacterial and fungal) disease outbreaks in aquaculture should be urgently considered, particularly in LMICs, either at the ecosystem or animal level. At the ecosystem level, the development of integrated farming and aquaculture practices is well known to improve ecosystem resilience, reduce organic matter inputs, and has been shown to increase disease resistance in farmed animals [51,54,55,56]. For example, in Vietnam, rice-fish farming has been widely adopted because ecological sustainability is improved through reduced pesticide use and increased nutrient recycling (less eutrophication) [55]. Such benefits have also been observed in Bangladesh [57],. Indonesia [58] and the Philippines [59]. Similarly, Shifflett and colleagues [54] have shown that the integration of highly productive forest plantations (via tree genotype selection) with freshwater aquaculture management can provide multi-functional landscapes, while efficiently producing fish protein, woody biomass, carbon sequestration, nitrogen storage, but also improved groundwater infiltration and reduced contaminant load in local surface waters, including pathogens. Indeed, while aquaculture often produces pond waters with high concentrations in nitrogen, phosphorous, organic carbon and chlorophyll a, leading to eutrophication (nutrient enrichment) when discharged into surface water and promoting the emergence of disease, the integration of aquaculture with managed forest plantations appears to be a more sustainable alternative to the current direct discharge of pond waters into rivers and streams [54].

At the level of livestock populations, genetic structuring, mixed species or multitrophic cropping systems aim to reduce the likelihood of EIDs and maximize ecosystem resilience to environmental and microbial challenges [2]. It would also be possible to improve biosecurity management measures and avoid the spread of pathogens, for example, by drying out culture units after each culture cycle, or even by domesticating broodstocks free of specific pathogen [17,21], although animals reared within open systems can get infected with local native pathogens.

Another alternative is based on vaccination, a promising method to reduce or avoid the use of chemical drugs in aquaculture, such as that previously used in Norwegian salmonids [60], although the design of vaccines remains a highly specific tool that targets one pathogen at a time, while coinfections with different pathogens are common in aquaculture facilities. In addition, vaccination can be a very expensive alternative for most farmers, especially in LMICs, and therefore may not be the best strategy for large-scale application [60,61,62].

Interestingly, the use of feed supplements could enhance the immune system of cultured aquatic animals and maximize fish growth and feeding efficiency [62,63,64]. For example, the use of prebiotics (inulin, fructooligosaccharides, short-chain fructooligosaccharides, mannanoligosaccharides, etc.), probiotics (the use of live micro-organisms, such as beneficial Gram-positive and Gram-negative bacteria) and bioactive plants is one of the most studied feed supplements in aquaculture to combat bacterial infections, for example, via immunostimulation in fish, shellfish and shrimp [61,64,65,66]. Some studies have already shown their beneficial effects on the physiology and immunity of aquatic animals in the fight against viral diseases [21]. For example, dietary administration of inulin (prebiotic) decreased the prevalence of WSSV in *L. vannamei* [67]. In another experiment, the combination of a prebiotic (isomaltooligosaccharides) and a probiotic (*Bacillus* OJ) enhanced immune response and disease resistance to *L. vannamei* to WSSV, while increasing its survival 14 days post-infection [68]. It has also been proposed that aquatic organisms can be immunostimulated by including pathogen-associated molecular patterns (PAMPs) to their feed, since PAMPs are known to activate the host innate immune system [21]. Reduced mortality of *P. japonicus* upon WSSV exposure was achieved by adding peptidoglycans derived from *Bifidobacterium thermophilum* to the feed, for instance [69], or by injecting β-glucan to the animals [70]. The use of medicinal plants in aquaculture has been widely reported to enhance animal growth, feeding efficiency, immune parameters and haematological parameters in fish, but also provide antioxidant activities, and was shown to increase disease resistance against several fish pathogens [62]. In West Java, Indonesia, herbal therapies commonly used in aquaculture are also plants commonly used in traditional human pharmacopeia, and are more commonly used in small farms than in large farming facilities [71]. The plants commonly used in these Indonesian farms belong to 79 species, 36 families and are commonly used to (i) improve water quality, (ii) reduce fish stress, (iii) enhance resistance to pathogens and (iv) treat fish diseases [71]. However, Reverter et al. (2020) [62] have shown that the use of low-cost alternatives for disease prevention alternatives, such as bioactive plants (either as power, plant extracts or essential oils), could indeed benefit small-scale rural farmers in LMICs, but also intensive farming facilities.

In LMICs, limited capacity and knowledge of pathogen surveillance and biosecurity remains a limitation. However, improved aquaculture health management skills and the application of a compensation policy [20] appear manageable and could significantly reduce the use of often expensive and harmful drugs, as well as the economic impact of (viral) disease outbreaks for aquaculture workers [61,65]. Overall, the future design of sustainable aquaculture systems worldwide should be considered from a One Health perspective in order to optimize ecosystem resilience, animal welfare and human well-being, supporting public health and food security on a global scale.

## 5. Conclusions and Perspectives

What our study has shown in the first instance is the lack of data on the relationship between temperature and mortality in fish and shellfish. However, as with bacteria [6], our results show the importance of characterizing the links between virus-related mortality and temperature. Here we have shown that even a relatively small temperature increase of 1 °C could lead to an increase in mortalities of 3–6%, which, in the context of LMIC aquaculture, could already have a significant impact on food safety. This study thus serves to raise awareness of the urgency of developing approaches to make aquaculture in LMICs (temperature mitigation, stock selection, health management, etc.) a sustainable source of protein for rapidly increasing populations.

## Figures and Tables

**Table 1:** Details from the selected linear models (LMs) and linear mixed models (LMMs) to test the relationship between aquatic animal mortality and temperature (T) under viral infections. The *p*-value is provided for LMs to indicate term significance. In LMMs, a term is considered significant when its 95% confidence interval does not include 0, and is indicated by *.

Data Subset	Model Selected	Adj. R^2^	Parameter	Estimate	SE	95% CI		*p*-Value	F-Value
						Lower	Upper		
Koi HV	Mortality ~ T + type of infection	0.408	T	4.77	1.09	2.55	6.98	<0.001	20.072
			Type of infection: injection	16.48	8.23	−0.28	33.23	0.054	4.002
Ostreid HV	Mortality ~ T	0.148	T	4.90	1.70	1.47	8.33	0.006	8.250
Betanodaviruses	Mortality ~ T + type of infection + (1|Host species) + (1|Pathogen.species)	0.719	T	3.78	0.79	2.18	5.37	*	20.051
			Life stage: larvae	47.35	11.67	22.97	72.00	*	6.799
			Type of infection: injection	20.579	5.63	9.29	31.80	*	
All viruses	Mortality ~ T + life stage + type of infection + (1|host species)	0.408	T	4.38	0.67	3.07	5.70	*	43.163
			Life stage: juvenile	−1.79	5.50	−12.83	9.20		1.769
			Life stage: larvae	31.79	13.57	4.16	61.23	*	
			Life stage: spat	3.31	13.29	−22.99	29.52		
			Type of infection: injection	11.40	4.80	1.87	20.89	*	5.631

## Supplementary Materials

The following supporting information can be downloaded at: https://www.mdpi.com/article/10.3390/microorganisms11041049/s1. References [72,73,74,75,76,77,78,79,80,81,82,83,84,85,86,87,88,89,90,91,92,93,94,95,96,97,98,99,100,101,102,103,104,105,106,107,108,109,110,111,112,113,114,115,116] are cited in the Supplementary Materials.
