# Supplementary material for: Impact of Global Warming on the Severity of Viral Diseases: A Potentially Alarming Threat to Sustainable Aquaculture Worldwide
Source: Microorganisms. 2023 Apr 17;11(4):1049. doi: 10.3390/microorganisms11041049 (PMC10146364; doi:10.3390/microorganisms11041049)

Supplementary Information

Supplementary Information S1. List of studies used in the present meta-analysis to extract data on temperature vs. cumulative mortalities of reared aquatic animals infected with viruses.

1. Badhusha, A.; Nafeez Ahmed, A.; Suryakodi, S.; Abdul Wazith, M.J.; Mithra, S.; Kanimozhi, K.; Abdul Majeed, S.; Taju, G.; Sahul Hameed, A.S. First report on the occurrence of cyprinid herpesvirus 3 in koi carp (Cyprinus carpio koi) in India. *J Fish Dis* **2022**, *45*, 1087-1098, doi:10.1111/jfd.13631.
2. Barsøe, S.; Skovgaard, K.; Sepúlveda, D.; Stratmann, A.; Vendramin, N.; Lorenzen, N. Nervous Necrosis Virus-like Particle (VLP) Vaccine Stimulates European Sea Bass Innate and Adaptive Immune Responses and Induces Long-Term Protection against Disease. *Pathogens* **2021**, *10*, doi:10.3390/pathogens10111477.
3. Bergmann, S.M.; Kempter, J. Detection of koi herpesvirus (KHV) after re-activation in persistently infected common carp (Cyprinus carpio L.) using non-lethal sampling methods. *Bulletin of the European Association of Fish Pathologists* **2011**, *31*, 92-100.
4. Bergmann, S.M.; Lutze, P.; Schütze, H.; Fischer, U.; Dauber, M.; Fichtner, D.; Kempter, J. Goldfish (Carassius auratus auratus) is a susceptible species for koi herpesvirus (KHV) but not for KHV disease (KHVD). *Bulletin of the European Association of Fish Pathologists* **2010**, *30*.
5. Bergmann, S.M.; Sadowski, J.; Kiełpiński, M.; Bartłomiejczyk, M.; Fichtner, D.; Riebe, R.; Lenk, M.; Kempter, J. Susceptibility of koi x crucian carp and koi x goldfish hybrids to koi herpesvirus (KHV) and the development of KHV disease (KHVD). *J Fish Dis* **2010**, *33*, 267-272, doi:10.1111/j.1365-2761.2009.01127.x.
6. Biasini, L.; Berto, P.; Abbadi, M.; Buratin, A.; Toson, M.; Marsella, A.; Toffan, A.; Pascoli, F. Pathogenicity of Different Betanodavirus RGNNV/SJNNV Reassortant Strains in European Sea Bass. *Pathogens* **2022**, *11*, doi:10.3390/pathogens11040458.
7. Burge, C.A.; Reece, K.S.; Dhar, A.K.; Kirkland, P.; Morga, B.; Dégremont, L.; Faury, N.; Wippel, B.J.T.; MacIntyre, A.; Friedman, C.S. First comparison of French and Australian OsHV-1 µvars by bath exposure. *Dis Aquat Organ* **2020**, *138*, 137-144, doi:10.3354/dao03452.
8. Chaves-Pozo, E.; Arizcun, M.; Cuesta, A. Betanodavirus genotypes produce clinical signs and mortality in the shi drum (Umbrina cirrosa), and infective particles are isolated from the damaged brain. *Aquaculture* **2021**, *541*, 736777, doi:https://doi.org/10.1016/j.aquaculture.2021.736777.
9. Corbeil, S.; Faury, N.; Segarra, A.; Renault, T. Development of an in situ hybridization assay for the detection of ostreid herpesvirus type 1 mRNAs in the Pacific oyster, Crassostrea gigas. *J Virol Methods* **2015**, *211*, 43-50, doi:10.1016/j.jviromet.2014.10.007.
10. Costes, B.; Raj, V.S.; Michel, B.; Fournier, G.; Thirion, M.; Gillet, L.; Mast, J.; Lieffrig, F.; Bremont, M.; Vanderplasschen, A. The major portal of entry of koi herpesvirus in Cyprinus carpio is the skin. *J Virol* **2009**, *83*, 2819-2830, doi:10.1128/jvi.02305-08.
11. de Kantzow, M.; Hick, P.; Becker, J.; Whittington, R. Effect of water temperature on mortality of Pacific oysters Crassostrea gigas associated with microvariant Ostreid herpesvirus-1 (OsHV-1 µVar). *Aquaculture Environment Interactions* **2016**, *8*, doi:10.3354/aei00186.
12. de Kantzow, M.C.; Whittington, R.J.; Hick, P. Prior exposure to Ostreid herpesvirus 1 (OsHV-1) at 18 °C is associated with improved survival of juvenile Pacific oysters (Crassostrea gigas) following challenge at 22 °C. *Aquaculture* **2019**, *507*, 443-450, doi:https://doi.org/10.1016/j.aquaculture.2019.04.035.
13. Dégremont, L.; Morga, B.; Maurouard, E.; Travers, M.-A. Susceptibility variation to the main pathogens of Crassostrea gigas at the larval, spat and juvenile stages using unselected and selected oysters to OsHV-1 and/or V. aestuarianus. *Journal of Invertebrate Pathology* **2021**, *183*, 107601, doi:https://doi.org/10.1016/j.jip.2021.107601.
14. Delisle, L.; Pauletto, M.; Vidal-Dupiol, J.; Petton, B.; Bargelloni, L.; Montagnani, C.; Pernet, F.; Corporeau, C.; Fleury, E. High temperature induces transcriptomic changes in Crassostrea gigas that hinder progress of ostreid herpesvirus (OsHV-1) and promote survival. *J Exp Biol* **2020**, *223*, doi:10.1242/jeb.226233.

15. Dong, C.; Li, X.; Weng, S.; Xie, S.; He, J. Emergence of fatal European genotype CyHV-3/KHV in mainland China. *Vet Microbiol* **2013**, *162*, 239-244, doi:10.1016/j.vetmic.2012.10.024.
16. Dong, C.; Weng, S.; Li, W.; Li, X.; Yi, Y.; Liang, Q.; He, J. Characterization of a new cell line from caudal fin of koi, Cyprinus carpio koi, and first isolation of cyprinid herpesvirus 3 in China. *Virus Research* **2011**, *161*, 140-149, doi:https://doi.org/10.1016/j.virusres.2011.07.016.
17. Evans, O.; Kan, J.Z.F.; Pathirana, E.; Whittington, R.J.; Dhand, N.; Hick, P. Effect of emersion on the mortality of Pacific oysters (Crassostrea gigas) infected with Ostreid herpesvirus-1 (OsHV-1). *Aquaculture* **2019**, *505*, 157-166, doi:https://doi.org/10.1016/j.aquaculture.2019.02.041.
18. Friedman, C.S.; Reece, K.S.; Wippel, B.J.T.; Agnew, M.V.; Dégremont, L.; Dhar, A.K.; Kirkland, P.; MacIntyre, A.; Morga, B.; Robison, C.; et al. Unraveling concordant and varying responses of oyster species to Ostreid Herpesvirus 1 variants. *Science of The Total Environment* **2020**, *739*, 139752, doi:https://doi.org/10.1016/j.scitotenv.2020.139752.
19. García-Álvarez, M.; Arizcun, M.; Chaves-Pozo, E.; Cuesta, A. Profile of Innate Immunity in Gilthead Seabream Larvae Reflects Mortality upon Betanodavirus Reassortant Infection and Replication. *Int J Mol Sci* **2022**, *23*, doi:10.3390/ijms23095092.
20. Gémez-Mata, J.; Souto, S.; Bandín, I.; Alonso, M.D.C.; Borrego, J.J.; Labella, A.M.; García-Rosado, E. Immune Response of Senegalese Sole against Betanodavirus Mutants with Modified Virulence. *Pathogens* **2021**, *10*, doi:10.3390/pathogens10111388.
21. Gilad, O.; Yun, S.; Adkison, M.A.; Way, K.; Willits, N.H.; Bercovier, H.; Hedrick, R.P. Molecular comparison of isolates of an emerging fish pathogen, koi herpesvirus, and the effect of water temperature on mortality of experimentally infected koi. *J Gen Virol* **2003**, *84*, 2661-2667, doi:10.1099/vir.0.19323-0.
22. Gye, H.J.; Oh, M.J.; Nishizawa, T. Lack of nervous necrosis virus (NNV) neutralizing antibodies in convalescent sevenband grouper Hyporthodus septemfasciatus after NNV infection. *Vaccine* **2018**, *36*, 1863-1870, doi:10.1016/j.vaccine.2018.02.063.
23. Hedrick, R.P.; Gilad, O.; Yun, S.; Spangenberg, J.V.; Marty, G.D.; Nordhausen, R.W.; Kebus, M.J.; Bercovier, H.; Eldar, A. A Herpesvirus Associated with Mass Mortality of Juvenile and Adult Koi, a Strain of Common Carp. *J Aquat Anim Health* **2000**, *12*, 44-57, doi:10.1577/1548-8667(2000)012<0044:Ahawmm>2.0.Co;2.
24. Hedrick, R.P.; Waltzek, T.B.; McDowell, T.S. Susceptibility of Koi Carp, Common Carp, Goldfish, and Goldfish × Common Carp Hybrids to Cyprinid Herpesvirus-2 and Herpesvirus-3. *Journal of Aquatic Animal Health* **2006**, *18*, 26-34, doi:https://doi.org/10.1577/H05-028.1.
25. Hu, F.; Li, Y.; Wang, Q.; Zhu, B.; Wu, S.; Wang, Y.; Zeng, W.; Yin, J.; Liu, C.; Bergmann, S.M.; et al. Immersion immunization of koi (Cyprinus carpio) against cyprinid herpesvirus 3 (CyHV-3) with carbon nanotube-loaded DNA vaccine. *Aquaculture* **2021**, *539*, 736644, doi:https://doi.org/10.1016/j.aquaculture.2021.736644.
26. Kim, Y.C.; Kwon, W.J.; Min, J.G.; Kim, K.I.; Jeong, H.D. Complete genome sequence and pathogenic analysis of a new betanodavirus isolated from shellfish. *J Fish Dis* **2019**, *42*, 519-531, doi:10.1111/jfd.12950.
27. Krishnan, R.; Jang, Y.S.; Kim, J.O.; Oh, M.J. Altered expression of immune factors in sevenband grouper, Hyporthodus septemfasciatus following nervous necrosis virus challenge at optimal and suboptimal temperatures. *Fish Shellfish Immunol* **2021**, *119*, 442-451, doi:10.1016/j.fsi.2021.10.033.
28. Morick, D.; Faigenbaum, O.; Smirnov, M.; Fellig, Y.; Inbal, A.; Kotler, M. Mortality Caused by Bath Exposure of Zebrafish (Danio rerio) Larvae to Nervous Necrosis Virus Is Limited to the Fourth Day Postfertilization. *Appl Environ Microbiol* **2015**, *81*, 3280-3287, doi:10.1128/aem.04175-14.
29. Nishizawa, T.; Gye, H.J.; Takami, I.; Oh, M.J. Potentiality of a live vaccine with nervous necrosis virus (NNV) for sevenband grouper Epinephelus septemfasciatus at a low rearing temperature. *Vaccine* **2012**, *30*, 1056-1063, doi:10.1016/j.vaccine.2011.12.033.
30. Oliver, R.; Fuhrmann, M.; Hick, P. Effect of air exposure, handling stress and imidacloprid on the susceptibility of Crassostrea gigas to Ostreid herpesvirus 1 (OsHV-1). *Aquaculture Environment Interactions* **2019**, *11*, 685-699.

31. Olveira, J.G.; Souto, S.; Dopazo, C.P.; Bandín, I. Isolation of betanodavirus from farmed turbot Psetta maxima showing no signs of viral encephalopathy and retinopathy. *Aquaculture* **2013**, *406-407*, 125-130, doi:https://doi.org/10.1016/j.aquaculture.2013.05.007.
32. Pannetier, P.; Morin, B.; Clérandeau, C.; Lacroix, C.; Cabon, J.; Cachot, J.; Danion, M. Comparative biomarker responses in Japanese medaka (Oryzias latipes) exposed to benzo[a]pyrene and challenged with betanodavirus at three different life stages. *Sci Total Environ* **2019**, *652*, 964-976, doi:10.1016/j.scitotenv.2018.10.256.
33. Pathirana, E.; Fuhrmann, M.; Whittington, R.; Hick, P. Influence of environment on the pathogenesis of Ostreid herpesvirus-1 (OsHV-1) infections in Pacific oysters (Crassostrea gigas) through differential microbiome responses. *Heliyon* **2019**, *5*, e02101, doi:10.1016/j.heliyon.2019.e02101.
34. Pathirana, E.; Whittington, R.J.; Hick, P.M. Impact of seawater temperature on the Pacific oyster (<i>Crassostrea gigas</i>) microbiome and susceptibility to disease associated with <i>Ostreid herpesvirus-1</i> (OsHV-1). *Animal Production Science* **2022**, *62*, 1040-1054, doi:https://doi.org/10.1071/AN21505.
35. Paul-Pont, I.; Evans, O.; Dhand, N.K.; Whittington, R.J. Experimental infections of Pacific oyster Crassostrea gigas using the Australian ostreid herpesvirus-1 (OsHV-1) µVar strain. *Dis Aquat Organ* **2015**, *113*, 137-147, doi:10.3354/dao02826.
36. Piačková, V.; Flajšhans, M.; Pokorová, D.; Reschová, S.; Gela, D.; Čížek, A.; Veselý, T. Sensitivity of common carp, Cyprinus carpio L., strains and crossbreeds reared in the Czech Republic to infection by cyprinid herpesvirus 3 (CyHV-3; KHV). *J Fish Dis* **2013**, *36*, 75-80, doi:10.1111/jfd.12007.
37. Picot, S.; Faury, N.; Pelletier, C.; Arzul, I.; Chollet, B.; Dégremont, L.; Renault, T.; Morga, B. Monitoring Autophagy at Cellular and Molecular Level in Crassostrea gigas During an Experimental Ostreid Herpesvirus 1 (OsHV-1) Infection. *Frontiers in Cellular and Infection Microbiology* **2022**, *12*, doi:10.3389/fcimb.2022.858311.
38. Qin, Y.; Liu, J.; Liu, W.; Shi, H.; Jia, A.; Lu, Y.; Liu, X. First isolation and identification of red-grouper nervous necrosis virus (RGNNV) from adult hybrid Hulong grouper (Epinephelus fuscoguttatus × Epinephelus lanceolatus) in China. *Aquaculture* **2020**, *529*, 735662, doi:https://doi.org/10.1016/j.aquaculture.2020.735662.
39. Rakus, K.; Irnazarow, I.; Adamek, M.; Palmeira, L.; Kawana, Y.; Hirono, I.; Kondo, H.; Matras, M.; Steinhagen, D.; Flasz, B.; et al. Gene expression analysis of common carp (Cyprinus carpio L.) lines during Cyprinid herpesvirus 3 infection yields insights into differential immune responses. *Dev Comp Immunol* **2012**, *37*, 65-76, doi:10.1016/j.dci.2011.12.006.
40. Schikorski, D.; Faury, N.; Pepin, J.F.; Saulnier, D.; Tourbiez, D.; Renault, T. Experimental ostreid herpesvirus 1 infection of the Pacific oyster Crassostrea gigas: Kinetics of virus DNA detection by q-PCR in seawater and in oyster samples. *Virus Research* **2011**, *155*, 28-34, doi:https://doi.org/10.1016/j.virusres.2010.07.031.
41. Schikorski, D.; Renault, T.; Saulnier, D.; Faury, N.; Moreau, P.; Pépin, J.-F. Experimental infection of Pacific oyster Crassostrea gigas spat by ostreid herpesvirus 1: demonstration of oyster spat susceptibility. *Veterinary Research* **2011**, *42*, 27, doi:10.1186/1297-9716-42-27.
42. Segarra, A.; Baillon, L.; Tourbiez, D.; Benabdelmouna, A.; Faury, N.; Bourgougnon, N.; Renault, T. Ostreid herpesvirus type 1 replication and host response in adult Pacific oysters, Crassostrea gigas. *Veterinary Research* **2014**, *45*, 103, doi:10.1186/s13567-014-0103-x.
43. Souto, S.; Olveira, J.G.; Bandín, I. Influence of temperature on Betanodavirus infection in Senegalese sole (Solea senegalensis). *Veterinary Microbiology* **2015**, *179*, 162-167, doi:https://doi.org/10.1016/j.vetmic.2015.07.004.
44. St-Hilaire, S.; Beevers, N.; Way, K.; Le Deuff, R.M.; Martin, P.; Joiner, C. Reactivation of koi herpesvirus infections in common carp Cyprinus carpio. *Dis Aquat Organ* **2005**, *67*, 15-23, doi:10.3354/dao067015.
45. Takahara, T.; Honjo, M.N.; Uchii, K.; Minamoto, T.; Doi, H.; Ito, T.; Kawabata, Z.i. Effects of daily temperature fluctuation on the survival of carp infected with Cyprinid herpesvirus 3. *Aquaculture* **2014**, *433*, 208-213, doi:https://doi.org/10.1016/j.aquaculture.2014.06.001.
46. Tan, T.L.; Paul-Pont, I.; Evans, O.M.; Watterson, D.; Young, P.; Whittington, R.; Fougerouse, A.; Bichet, H.; Barnes, A.C.; Dang, C. Resistance of Black-lip learl oyster, Pinctada

margaritifera, to infection by Ostreid herpes virus 1µvar under experimental challenge may be mediated by humoral antiviral activity. *Fish Shellfish Immunol* **2015**, *44*, 232-240, doi:10.1016/j.fsi.2015.02.026.
47. Toffan, A.; De Salvador, M.; Scholz, F.; Pretto, T.; Buratin, A.; Rodger, H.D.; Toson, M.; Cuenca, A.; Vendramin, N. Lumpfish (Cyclopterus lumpus, Linnaeus) is susceptible to viral nervous necrosis: Result of an experimental infection with different genotypes of Betanodavirus. *Journal of Fish Diseases* **2019**, *42*, 1667-1676, doi:https://doi.org/10.1111/jfd.13088.
48. Toffan, A.; Panzarin, V.; Toson, M.; Cecchettin, K.; Pascoli, F. Water temperature affects pathogenicity of different betanodavirus genotypes in experimentally challenged Dicentrarchus labrax. *Dis Aquat Organ* **2016**, *119*, 231-238, doi:10.3354/dao03003.
49. Vaz, M.; Pires, D.; Pires, P.; Simões, M.; Pombo, A.; Santos, P.; do Carmo, B.; Passos, R.; Costa, J.Z.; Thompson, K.D.; et al. Early Immune Modulation in European Seabass (Dicentrarchus labrax) Juveniles in Response to Betanodavirus Infection. *Fishes* **2022**, *7*, 63.
50. Vázquez-Salgado, L.; Olveira, J.G.; Dopazo, C.P.; Bandín, I. Effect of rearing density on nervous necrosis virus infection in Senegalese sole (Solea senegalensis). *Journal of Fish Diseases* **2021**, *44*, 2003-2012, doi:https://doi.org/10.1111/jfd.13514.
51. Yamashita, H.; Mori, K.; Kuroda, A.; Nakai, T. Neutralizing antibody levels for protection against betanodavirus infection in sevenband grouper, Epinephelus septemfasciatus (Thunberg), immunized with an inactivated virus vaccine. *J Fish Dis* **2009**, *32*, 767-775, doi:10.1111/j.1365-2761.2009.01054.x.
52. Yuasa, K.; Ito, T.; Sano, M. Effect of Water Temperature on Mortality and Virus Shedding in Carp Experimentally Infected with Koi Herpesvirus. *Fish Pathology* **2008**, *43*, 83-85, doi:10.3147/jsfp.43.83.

Supplementary Information S2. Comparison and performance ranking of linear models (LMs) to test the effect of temperature (T) on the mortality of oysters infected with Ostreid herpesvirus (OsHv-1). Specific subset 1 (SSb1).

All studies included here were conducted on *Crassostrea gigas* and therefore no evaluation of random effects was necessary. Fixed effects included in the model were temperature, type of infection, life stage and log(dose).

Model ranking	Model	df	AICc	ΔAICc	w_i
Selection of fixed effects (linear regression model)					
**1**	**Mortality ~ T**	**3**	**477.0**	**0.00**	**0.401**
2	Mortality ~ T + type of infection*log(dose)	6	478.6	1.62	0.179
3	Mortality ~ T + type of infection	4	478.8	1.78	0.165
4	Mortality ~ T + log(dose)	4	479.3	2.26	0.129
5	Mortality ~ T + type of infection + log(dose)	5	481.1	4.09	0.052
6	Mortality ~ T + life stage	5	481.9	4.85	0.036
7	Mortality ~ T + life stage + type of infection	6	483.8	6.81	0.013
8	Mortality ~ T + type of infection*log(dose) + life stage	8	484.2	7.19	0.011
9	Mortality ~ T + life stage + log(dose)	6	484.4	7.33	0.010
10	Mortality ~ T + type of infection + log(dose) + life stage	7	486.4	9.38	0.004

Supplementary Information S3. Comparison and performance ranking of linear models (LMs) to test the effect of temperature (T) on the mortality of cyprinid fishes infected with Koi herpesvirus (KHV – Cyp-HV-3). Specific subset 2 (SSb2). The highest ranking model (in bold) was selected for further study.

All studies included here were conducted on *Cyprinus carpio* and therefore no evaluation of random effects was necessary. Fixed effects included in the model were temperature, type of infection, life stage and log(dose).

Model ranking	Model	df	AICc	ΔAICc	w_i
Selection of fixed effects (linear regression model)					
**1**	**Mortality ~ T + type of infection**	**4**	**382.8**	**0.00**	**0.407**
2	Mortality ~ T	3	330.4	1.58	0.185
3	Mortality ~ T + life stage + type of infection	5	331.3<br>331.4	2.57	0.113
4	Mortality ~ T + type of infection + log(dose)	5	332.7	2.63	0.109
5	Mortality ~ T + life stage	4	332.9	3.97	0.056
6	Mortality ~ T + log(dose)	4	334.1	4.12	0.052
7	Mortality ~ T + time of infection*log(dose)	6	334.2	5.28	0.029
8	Mortality ~ T + life stage + type of infection + log(dose)	6	335.4	5.40	0.027
9	Mortality ~ T + life stage +log(dose)	5	336.9	6.67	0.014
10	Mortality ~ T + life stage + type of infection*log(dose)	7		8.12	0.007

Supplementary Information S4. Comparison and performance ranking of linear models (LMMs) to test the effect of temperature (T) on the mortality of fishes infected with betanodavirus (RGNNV, KSNNV, SGNNV, BFNNV and TPNNV). Specific subset 3 (SSb3). The highest ranking model (in bold) was selected for further study.

Random effects included in the model were host family, host species, and pathogen species. Fixed effects included in the model were temperature, type of infection, life stage and log(dose).

Model ranking	Model	df	AICc	ΔAICc	w_i
**Selection of random effects**					
1	Mortality ~ T + (1 | Host species) + (1 | Pathogen species)	5	717.2	0.00	0.310
2	Mortality ~ T + (1 | Host species)	4	717.8	0.60	0.230
3	Mortality ~ T + (1 | Pathogen species) + (1 | Host family)	5	718.5	1.34	0.158
4	Mortality ~ T + (1 | Host family)	4	718.9	1.69	0.134
5	Mortality ~ T + (1 | Pathogen species) + (1 | Host family/species)	6	719.5	2.37	0.095
6	Mortality ~ T + (1 | Host family/ Host species)	5	720.1	2.90	0.073
7	Mortality ~ T + (1 | Pathogen species)	4	732.0	14.87	0.000
8	Mortality ~ T	3	741.6	24.39	0.000
**Selection of fixed effects (linear regression model)**					
**1**	**Mortality ~ T + life stage + type of infection + (1 | Host species) + (1 | Pathogen.species)**	**7**	**702.1**	**0.00**	**0.481**
2	Mortality ~ T + type of infection*log(dose) + life stage + (1 | Host species) + (1 | Pathogen species)	9	703.3	1.21	0.263
3	Mortality ~ T + type of infection + log(dose) + life stage + (1 | Host species) + (1 | Pathogen species)	8	704.1	2.00	0.177
4	Mortality ~ T + type of infection*log(dose) + (1 | Host species) + (1 | Pathogen species)	8	706.0	3.94	0.067
5	Mortality ~ T + life stage + (1 | Host species) + (1 | Pathogen species)	6	711.6	9.48	0.004
6	Mortality ~ T + log(dose) + life stage + (1 | Host species) + (1 | Pathogenspecies)	7	712.1	10.02	0.003
7	Mortality ~ T + type of infection + log(dose) + (1 | Host species) + (1 | Pathogen species)	7	712.8	10.74	0.002
8	Mortality ~ T + type of infection + (1 | Host species) + (1 | Pathogen species)	6	713.4	11.35	0.002
9	Mortality ~ T + log(dose)+ (1 | Host species) + (1 | Pathogen species)	6	715.7	15.57	0.001
10	Mortality ~ T + (1 | Host species) + (1 | Pathogen species)	5	717.2	15.08	0.000

Supplementary Information S5. Comparison and performance ranking of linear mixed models (LMMs) to test the effect of temperature (T) on the mortality host species infected by viruses. General subset 1 (GSb1). Random effects included host species, host family,pathogen species and family and fixed effects included temperature, type of infection, life stage and log(dose). The highest ranking model (in bold) was selected for further study.

Model ranking	Model	df	AICc	ΔAICc	w_i
Selection of random effects					
1	Mortality ~ T + (1 | host species)	4	1523.8	0.00	0.246
2	Mortality ~ T + (1 | host species) + (1 | pathogen species)	5	1524.2	0.48	0.193
3	Mortality ~ T + (1 | host family)	4	1525.8	2.08	0.087
4	Mortality ~ T + (1 | host species) + (1 | pathogen family)	5	1525.9	2.13	0.085
5	Mortality ~ T + (1 | host family/species)	5	1525.9	2.13	0.085
6	Mortality ~ T + (1 | pathogen species) + (1 | host family)	5	1526.2	2.50	0.070
7	Mortality ~ T + (1 | host family/species) + (1 | pathogen species)	6	1526.4	2.64	0.065
8	Mortality ~ T + (1 | pathogen family/species) + (1 | host species)	6	1526.4	2.64	0.065
9	Mortality ~ T + (1 | host family) + (1 | pathogen family)	5	1528.0	4.21	0.030
10	Mortality ~ T + (1 | pathogen family) + (1 | host family/species)	6	1528.0	4.29	0.029
11	Mortality ~ T + (1 | pathogen family/species) + (1 | host family)	6	1528.4	4.66	0.024
12	Mortality ~ T + (1 | pathogen family/species) + (1 | host family/species)	7	1528.6	4.83	0.022
13	Mortality ~ T + (1 | pathogen species)	4	1540.6	16.80	0.000
14	Mortality ~ T + (1 | pathogen family/species)	5	1542.7	18.94	0.000
15	Mortality ~ T + (1 | pathogen family)	4	1558.8	35.02	0.000
16	Mortality ~ T +	3	1587.3	63.52	0.000
Selection of fixed effects (linear regression model)					
**1**	**Mortality ~ T + life stage + type of infection + (1 | host species)**	**8**	**1522.2**	**0.00**	**0.246**
2	Mortality ~ T + type of infection + (1 | host species)	5	1522.6	0.45	0.197
3	Mortality ~ T ++ type of infection*log(dose) + (1 | host species)	7	1523.5	1.39	0.123
4	Mortality ~ T + (1 | host species)	4	1523.8	1.59	0.111
5	Mortality ~ T + type of infection + log(dose) + life stage + (1 | host species)	9	1524.4	2.25	0.080
6	Mortality ~ T + type of infection + log(dose) + (1 | host species)	6	1524.6	2.43	0.073
7	Mortality ~ T + life stage + type of infection*log(dose) + (1 | host species)	10	1525.0	2.87	0.059
8	Mortality ~ T + life stage + (1 | host species)	7	1525.4	3.25	0.049
9	Mortality ~ T + log(dose) + (1 | host species)	5	1525.6	3.41	0.045
10	Mortality ~ T + log(dose) + life stage + (1 | host species)	8	1527.5	5.38	0.017

Supplementary Information S6: Diagnostic plots (residual plot, QQ plot and partial autocorrelation plot) of selected models. A: Plots for OsHV-1 model. B: plots for KHV model. C: plots for NVVs model. D: plots for all viruses model.

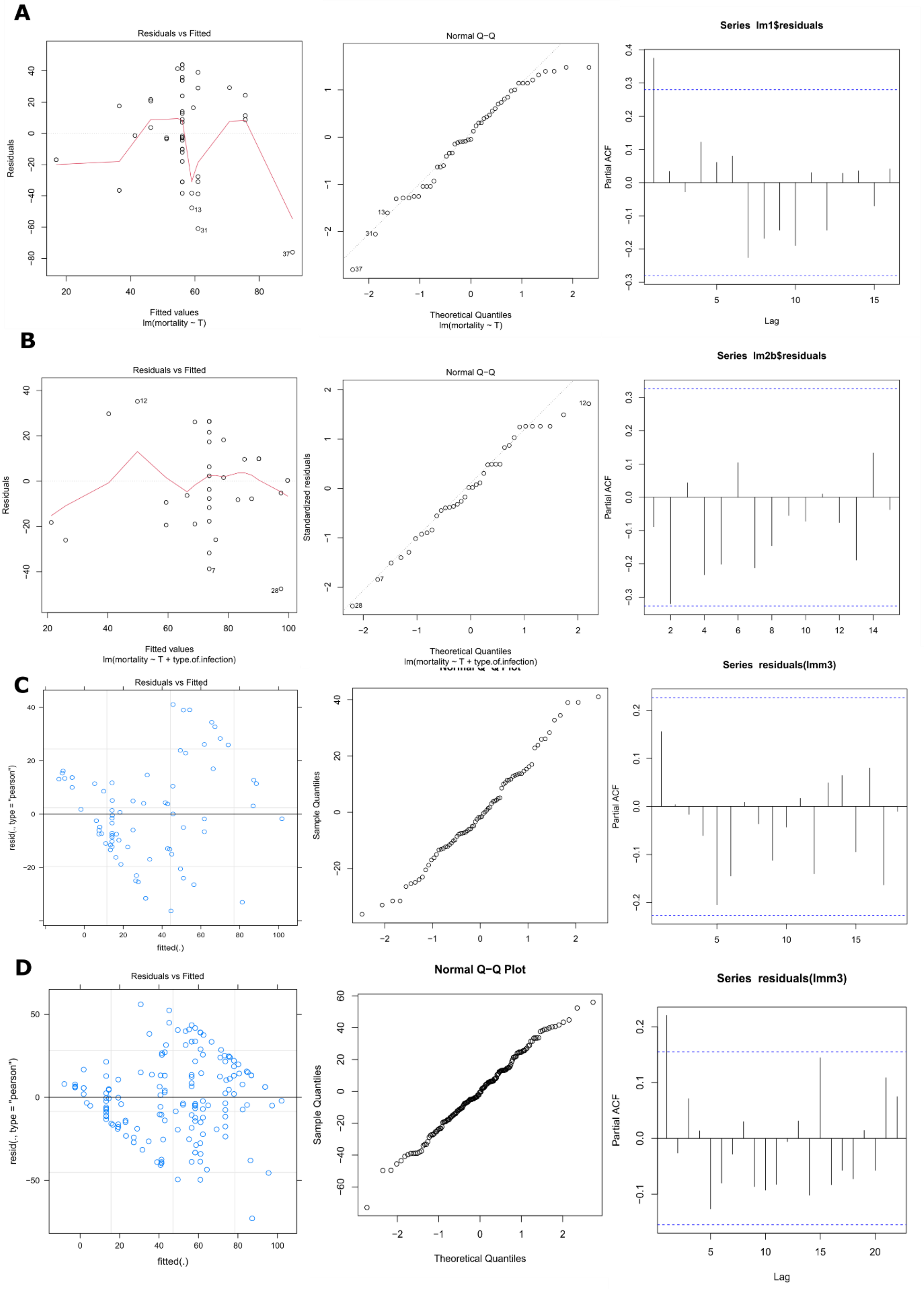

Supplement: Supplementary file 1 [file microorganisms-11-01049-s001.zip › microorganisms-2244204-supplementary.pdf]
